# *Coccidioides immitis* Cervical Lymphadenitis Complicated by Esophageal Fistula

**DOI:** 10.1155/2016/8715405

**Published:** 2016-05-23

**Authors:** Michael Loudin, Daniel R. Clayburgh, Morgan Hakki

**Affiliations:** ^1^Department of Internal Medicine, Oregon Health and Science University, Portland, OR 97239, USA; ^2^Department of Otolaryngology-Head and Neck Surgery, Oregon Health and Science University, Portland, OR 97239, USA; ^3^Division of Infectious Diseases, Oregon Health and Science University, Portland, OR 97239, USA

## Abstract

Coccidioidomycosis (valley fever) is caused by the dimorphic fungi* Coccidioides immitis* or* Coccidioides posadasii*. Most infections are asymptomatic or result in self-limited pneumonia; extrapulmonary dissemination via either hematogenous or lymphatic spread is rare. Here, we present a case of cervical* C. immitis* lymphadenitis that resulted in fistula formation to the esophagus via mediastinal extension. This case highlights a very unusual extrapulmonary manifestation of coccidioidomycosis, the difficulty in diagnosing coccidioidal infection when it is not suspected, and the importance of obtaining a thorough exposure history to assist with diagnosis.

## 1. Introduction

The disease coccidioidomycosis (valley fever) results from inhaling the spores of* Coccidioides immitis* or* Coccidioides posadasii* [1]. While the vast majority of infections either are asymptomatic or result in self-limited pneumonia, extrapulmonary disease, most commonly of the skin, bone and joints, or meninges, complicates approximately 0.5% of infections in the general population [1–3]. Supraclavicular and cervical lymphadenopathy may result from lymphatic drainage of a primary pulmonary infectious source [1] or present as the only sign of coccidioidomycosis in patients without pulmonary disease [4]. We report the first example, to the best of our knowledge, of* C. immitis* cervical lymphadenitis associated with fistula formation to the esophagus. In addition to this unusual manifestation of* C. immitis* infection, this case illustrates the difficulty in diagnosing coccidioidal infection when it is not suspected and the importance of obtaining a thorough exposure history to assist with diagnostic evaluation.

## 2. Case Presentation

An 82-year-old male with a past medical history significant for a 55-pack-year history of smoking, hypertension, coronary artery disease, and systolic heart failure presented to his local medical providers with a nontender mass in his right neck of 2-3 weeks' duration. He also noted three months' worth of fatigue, poor appetite, night sweats, and a 30-pound weight loss. As per clinical documentation, examination at that time was notable for a 5-6 cm right supraclavicular mass with several areas of apparent subcutaneous purulence. Fine needle aspiration (FNA) of the neck mass yielded purulent material; bacterial and mycobacterial cultures were negative. Over the following month, he was treated with several courses of oral antibacterial agents without benefit. During this time, he developed a chronically draining cutaneous sinus tract from the neck mass productive of purulent material. Two separate swabs of the drainage material submitted for bacterial, mycobacterial, and fungal cultures were negative. An ultrasound-guided core biopsy of the mass revealed focal noncaseating granulomas but no cultures were sent; stains for bacteria (Gram stain), acid fast bacilli (Fite's stain), and fungi (Gomori methenamine silver, GMS) were negative. Broad-range 16s ribosomal RNA (rRNA) polymerase chain reaction (PCR) performed on paraffin-embedded tissue obtained at the time of core biopsy was negative for bacterial, fungal, or mycobacterial DNA. Given the uncertainty surrounding the diagnosis, lack of benefit from previous antibacterial agents, and apparent clinical stability, no further treatment was given at this time.

Approximately 7 months after onset of illness, he was admitted to his local hospital for ongoing fevers, night sweats, weight loss, and drainage from his neck lesion, along with progressive dysphagia and a dry cough that had developed over the preceding 1-2 months. At the time of admission, it was noted that his right neck mass had continued to drain “brownish” fluid but had not changed appreciably. CT scan of the chest demonstrated a collection of gas within the upper right mediastinum with apparent communication to the esophagus concerning for esophageal perforation and a small focal infiltrate in the upper right lung (not shown).

**Figure 1:**
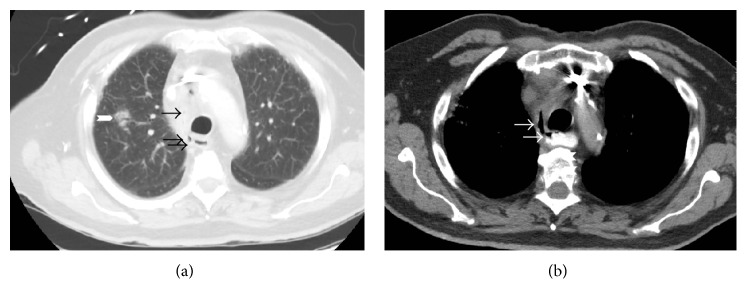
(a) Sagittal CT scan demonstrating fistulous tract containing air bubbles (arrows) and surrounding inflammation extending from the right lateral esophageal wall and right upper lobe pulmonary opacification (chevron). (b) CT esophagram demonstrating fistulous tract (arrows) extending from the right anterolateral esophagus to the mediastinum.

**Figure 2:**
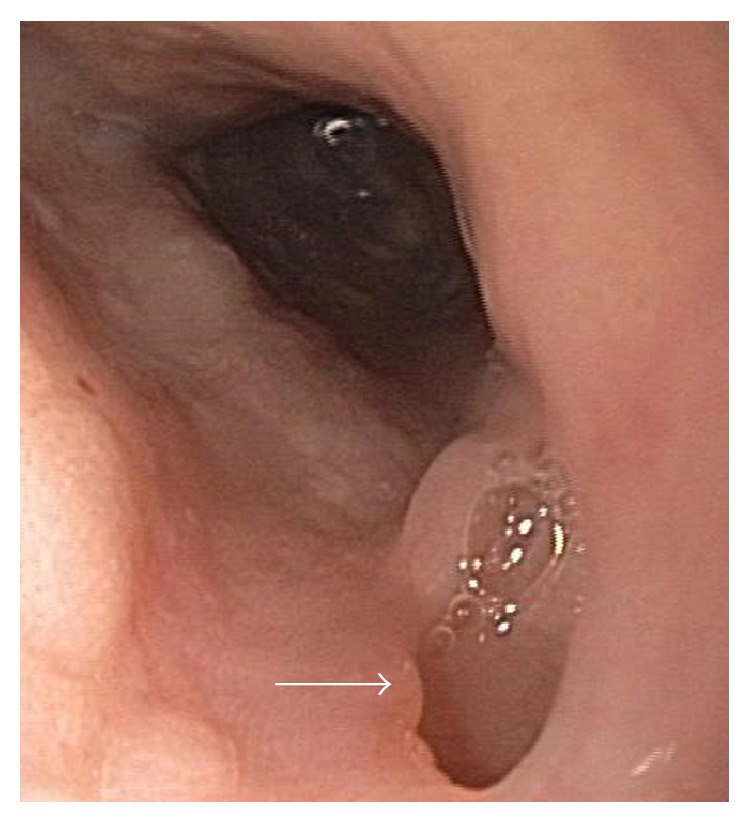
Esophagoduodenoscopic image demonstrating a chronic-appearing, well-epithelialized fistulous tract (arrow) at approximately 24 cm (8 cm distal to upper esophageal sphincter). Image courtesy of Dr. Gene Bakis.

At this point, he was transferred to our institution for management of suspected esophageal perforation. Repeat CT of the chest demonstrated right upper lobe pulmonary nodular consolidation in addition to soft tissue inflammation within the right thoracic inlet/supraclavicular area extending to the lower anterior right neck with associated mild right hilar and mediastinal lymph node enlargement (Figures 1(a) and 1(b)). Additionally, an apparent fistulous tract was noted, originating from the right lateral esophageal wall, coursing around the trachea, and reaching towards the mid mediastinum (Figures 1(a) and 1(b)). Esophagoduodenoscopy revealed a small pinhole opening in the esophagus surrounded by granulation tissue (Figure 2) that was sealed using fibrin glue. His hospital course was uneventful and he was discharged once tolerating oral intake. The neck lesion was not evaluated during that hospitalization.

**Figure 3:**
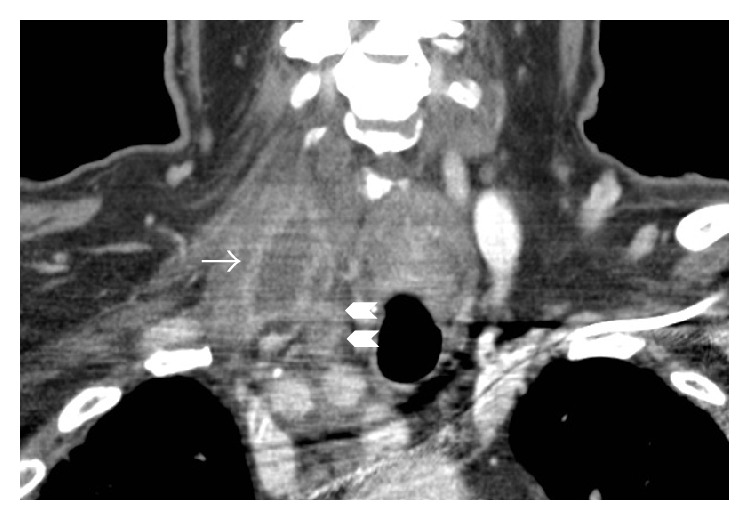
Coronal CT demonstrating a rim-enhancing fluid collection in the right neck (arrow) and inflammatory changes tracking into the mediastinum (chevrons).

Three months later, approximately 10 months after illness onset, he presented to our institution's Head and Neck Surgery Center and Infectious Diseases Clinic with ongoing purulent drainage and swelling at his right neck, fevers, night sweats, and continued weight loss. CT of the neck showed a 2.4 × 2.2 × 3.8 cm rim-enhancing fluid collection involving the inferior aspect of the right neck just anterior to the right first rib with inflammation tracking into the mediastinum (Figure 3). He underwent right selective neck dissection with excision of an extremely friable and inflamed tissue mass from level IV of the neck. Pathologic examination of the mass revealed necrosis and necrotizing granuloma formation within the soft tissue, with rare granulomas present in an adjacent lymph node. Pathology of the portion of the sinus tract that was excised similarly demonstrated necrosis and necrotizing granuloma formation. Stains for bacteria (Gram stain), fungi (GMS), and acid fast bacilli (Fite's stain) were performed on tissue containing granulomatous inflammation and were all negative.

Fungal culture from the excised tissue mass grew* Coccidioides immitis* that was confirmed by DNA probe. Complement fixation performed on serum (University of California, Davis) was positive at a titer of 1 : 8 (IgG); precipitin (IgM) was negative. Upon further questioning, he reported spending winters in Arizona for approximately 10 years and reported a history of self-limited valley fever approximately 6 years prior to this presentation. At the time of postoperative evaluation, he continued to have drainage from his neck at the inferior/proximal aspect of his surgical incision.

Oral fluconazole therapy at 400 mg daily was initiated. After two months of therapy, he self-discontinued fluconazole due to intolerable gastrointestinal side effects and severe generalized pruritis. During this time, he did not gain any weight due to poor appetite and overall did not feel any better. However, the amount of drainage from his neck sinus tract decreased, and his fevers and night sweats abated. Once he stopped fluconazole, he felt better almost immediately, with resolution of pruritis and gastrointestinal side effects. He declined itraconazole and instead elected to take a break from antifungal therapy. Two months after discontinuation of antifungal therapy, his appetite had improved and he had regained weight, and the drainage from his neck sinus tract continued to decrease nearly to the point of complete resolution. Serial follow-up CT scans demonstrated evolution of the right upper lobe pulmonary opacity into a more solid lesion without appreciable change in size, which raised concern for primary lung adenocarcinoma. He declined immediate resection of the affected lung, preferring to be followed up radiographically for the time being.

## 3. Discussion

Endemic to southern Arizona, central California, southern New Mexico, and west Texas in the United States,* C. immitis* may cause self-limited pneumonia, although the majority of infections are asymptomatic [1]. Pulmonary sequelae such as nodules or cavities occur in a minority (5% or less) of cases [2]. Extrapulmonary disease complicates approximately 0.5% of infections in the general population [1, 3]. Persons at greater risk for disseminated disease include immunocompromised hosts, men, pregnant women, and persons of Filipino or African ancestry [1, 3]. The most common sites of dissemination are the skin, bone and joints, and meninges [3].

Supraclavicular and cervical lymphadenopathy may result from direct lymphatic drainage of a primary pulmonary infectious source [1] or manifestation of disseminated disease [5] or as the only sign of coccidioidomycosis in patients without pulmonary disease [4]. It is not clear that our patient had a primary pulmonary source, as was initially suspected based on initial imaging findings, since the evolution of the lung lesion appears to be more consistent with a primary lung adenocarcinoma. As of the submission of this report, he has not undergone a definitive diagnostic procedure for his evolving lung lesion. In addition to lymph node involvement, infection of other head and neck structures including retropharyngeal abscess and infection of the thyroid, larynx, and trachea have been described [6]. However, the incidence of head and neck disease, with or without associated pulmonary disease, is not well defined; case reports are limited in number [4, 7–11].

To the best of our knowledge, esophageal fistula formation associated with cervical lymphadenitis has not been a previously reported complication of coccidioidomycosis. Indeed, this appears to be a very rare complication of cervical lymphadenitis of any infectious etiology, with only one case secondary to tuberculosis cervical lymphadenitis being reported [12]. While a fistulous communication from the neck to the esophagus via mediastinal extension of infection from the primary cervical lymph node source was not formally documented by a radiographic study, one was almost certainly present based on the combination of CT scan and esophagoduodenoscopy findings and the clinical presentation.

Establishing the diagnosis of* C. immitis* cervical lymphadenitis can be challenging. Culture of FNA samples from an involved lymph node may yield the organism [5, 10] but is often negative [6]; excisional biopsy appears to have a higher yield [6]. Characteristic histopathologic findings on biopsy with [4] or without [7] supporting serologic testing may also be used. In our patient, neither fungal cultures of drainage material nor PCR of a core biopsy specimen yielded* C. immitis*. Instead, culture of a larger tissue volume obtained at the time of mass excision was required. Information about his part-time residence in an endemic area and history of prior valley fever were obtained subsequent to this finding. Knowledge of these aspects of his background prior to this may have altered our fundamental approach to diagnostics and interventional procedures.

The optimal management in this setting is not clear. Insufficient clinical experience exists as to whether medical therapy alone will be sufficient to result in permanent closure of the esophageal fistula over the long term, as has been reported in the successful management of tuberculous fistulae [12], or whether surgical repair of the fistula will eventually be required. For cervical lymphadenitis without the complicating fistula, there are reports of patients who have been treated successfully with systemic antifungal medication alone and in conjunction with either needle aspiration or surgical excision to reduce the fungal burden of infection [5]. Excision of the majority of infected tissue was performed in this case for both diagnostic and therapeutic purposes. That our patient continues to improve two months after having discontinued what amounted to a relatively short eight-week course of therapy may indicate that prolonged antifungal therapy and surgical takedown of the fistula may not be required.

## 4. Conclusions

This case represents a very unusual, previously unreported, clinical complication of* C. immitis* infection. Recognition of risk factors for* C. immitis* infection based on exposure history is a critical aspect of making a timely diagnosis and guiding the approach to therapy.

## References

[1] Galgiani J., Mandell G. L., Bennett J. E., Dolin R. (2005). Coccidioides species. *Principles and Practice of Infectious Diseases*.

[2] Galgiani J. N., Ampel N. M., Blair J. E. (2005). Coccidioidomycosis. *Clinical Infectious Diseases*.

[3] Stevens D. A. (1995). Current concepts: coccidioidomycosis. *The New England Journal of Medicine*.

[4] Dudley J. E. (1987). Coccidioidomycosis and neck mass “single lesion” disseminated disease. *Archives of Otolaryngology-Head and Neck Surgery*.

[5] Biller J. A., Scheuller M. C., Eisele D. W. (2004). Coccidioidomycosis causing massive cervical lymphadenopathy. *Laryngoscope*.

[6] Copeland B., White D., Buenting J. (2003). Coccidioidomycosis of the head and neck. *Annals of Otology, Rhinology and Laryngology*.

[7] D'Avino A., Di Giambenedetto S., Fabbiani M., Farina S. (2012). Coccidioidomycosis of cervical lymph nodes in an HIV-infected patient with immunologic reconstitution on potent HAART: a rare observation in a nonendemic area. *Diagnostic Microbiology and Infectious Disease*.

[8] Hicks M. J., Green L. K., Clarridge J. (1994). Primary diagnosis of disseminated coccidioidomycosis by fine needle aspiration of a neck mass: a case report. *Acta Cytologica*.

[9] Kafka J. A., Catanzaro A. (1981). Disseminated coccidioidomycosis in children. *The Journal of Pediatrics*.

[10] Mathew G., Smedema M., Wheat L. J., Goldman M. (2003). Relapse of coccidioidomycosis despite immune reconstitution after fluconazole secondary prophylaxis in a patient with AIDS. *Mycoses*.

[11] Newland Y., Komisar A. (1986). Coccidioidomycosis of the head and neck. *Ear, Nose and Throat Journal*.

[12] Cataño J., Cardeño J. (2013). Perforated tuberculosis lymphadenitis. *The American Journal of Tropical Medicine and Hygiene*.

